# Laparoscopic left lateral sectionectomy for a patient with right-sided ligamentum teres

**DOI:** 10.1186/s40792-019-0601-1

**Published:** 2019-03-15

**Authors:** Fumihiro Terasaki, Yusuke Yamamoto, Katsuhisa Ohgi, Teiichi Sugiura, Yukiyasu Okamura, Takaaki Ito, Ryo Ashida, Katsuhiko Uesaka

**Affiliations:** Division of Hepato-Biliary-Pancreatic Surgery, Shizuoka Cancer Center, 1007 Shimonagakubo, Nagaizumi-cho, Sunto-gun, Shizuoka 411-8777 Japan

**Keywords:** Laparoscopic hepatectomy, Right-sided ligamentum teres, Liver metastasis, Left lateral sectionectomy, Left-sided gallbladder

## Abstract

**Background:**

A right-sided ligamentum teres (RSLT) is a rare congenital anomaly in which the fetal umbilical vein is connected to the right paramedian trunk. RSLT creates difficulty in liver resection with respect to decision-making regarding the resection line, deviation of the vasculobiliary architecture. We report a case in which laparoscopic left lateral sectionectomy (LLLS) was performed to treat colorectal liver metastasis (CRLM) in a patient with RSLT.

**Case presentation:**

A 63-year-old man with a past history of rectal cancer presented to our institution due to liver metastasis in the left lateral section from rectal cancer. In this patient, an RSLT was diagnosed and LLLS was planned. The lateral superior branch of the portal vein (P2) branched off behind the bifurcation of the portal vein and running separately from the common branch of the lateral inferior branch (P3) and left paramedian branch (P4) so that stapling could not be performed for liver resection. Frequent intraoperative ultrasonography (IOUS) was necessary to identify the root of P2 and P3. The resection line was distant from the falciform ligament and was carefully decided. The lateral superior branch of Glisson (G2) and lateral inferior branch of Glisson (G3) were separately resected. The patient had a favorable clinical course without any complications.

**Conclusions:**

The resection line of LLLS, which is distant from the falciform ligament, should be carefully identified using IOUS due to the deviation of the umbilical portion and falciform ligament. The recognition of portal vein and hepatic vein anomalies and clear identification of the lateral sectional branches are important to complete LLLS in patients with an RSLT.

## Background

A right-sided ligamentum teres (RSLT) is a rare congenital anomaly defined by the fetal umbilical vein being connected to the right paramedian trunk, with a reported prevalence of 0.1–1.2% [1, 2]. The anomaly was first reported by Hochstetter et al. in 1886 [3]. An RSLT causes difficulty in laparoscopic left lateral sectionectomy (LLLS) with respect to decision-making regarding the resection line, which is far from the falciform ligament, deviation of the vascular and biliary architecture, and is disadvantageous for performing stapling. We herein report a case in which LLLS was performed to treat colorectal liver metastasis (CRLM) in a patient with an RSLT.

## Case presentation

**Fig. 1:**
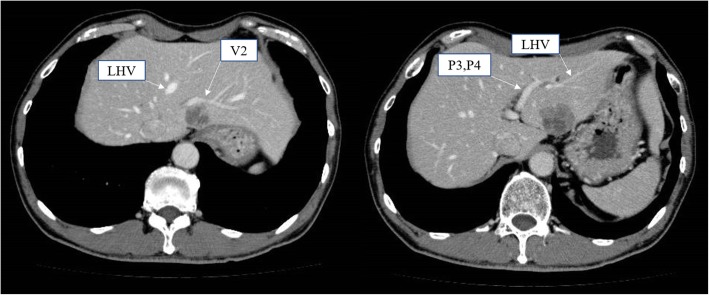
Abdominal contrast-enhanced CT revealed a hypovascular tumor in the left lateral section. V2, drainage vein of the lateral superior section; LHV, left hepatic vein; P3, lateral inferior branch of the portal vein; P4, left paramedian branch of the portal vein

**Fig. 2:**
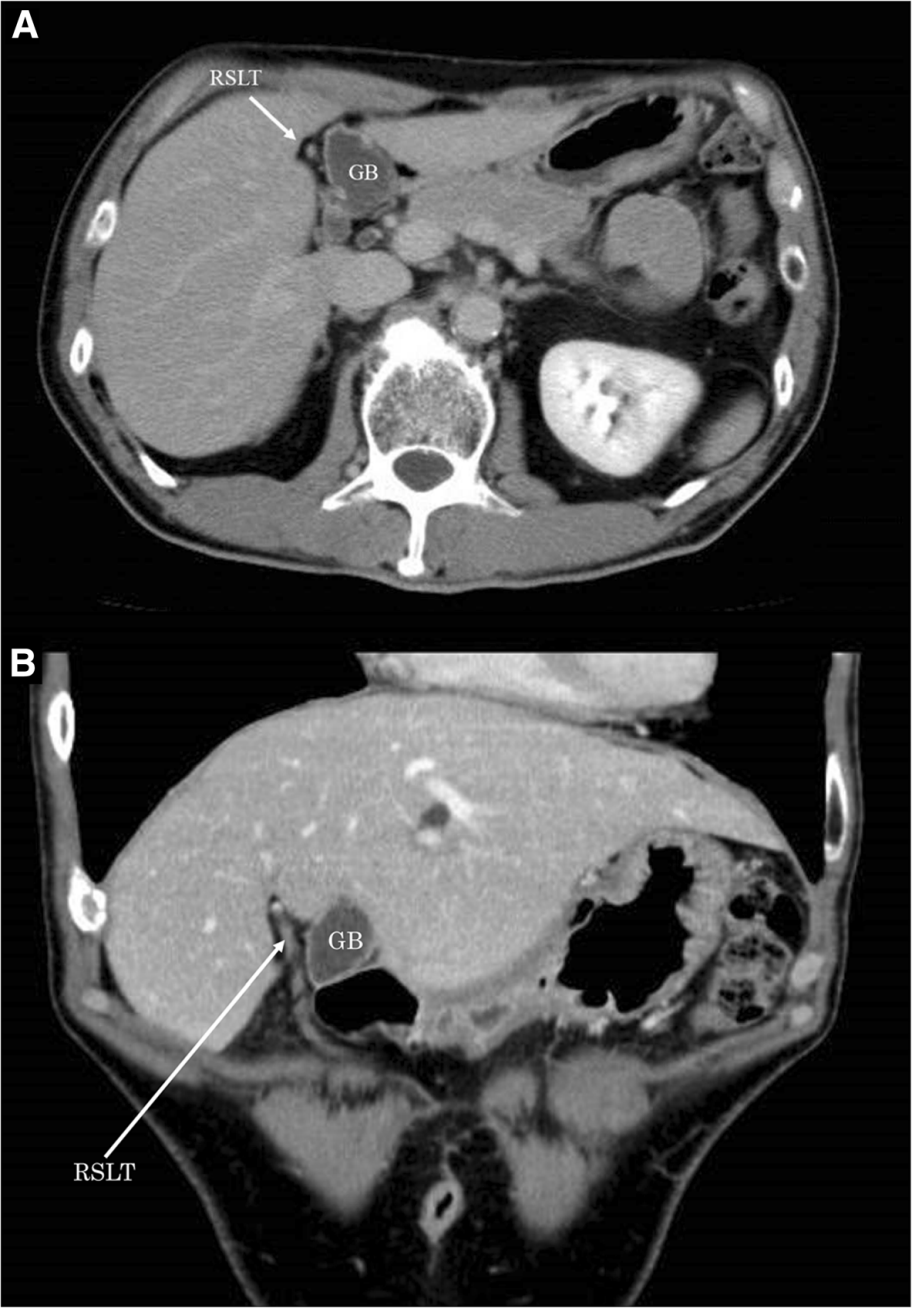
**a** Axial contrast-enhanced CT showed the ligamentum teres observed on the right side of the gallbladder (arrow). GB, gallbladder; RSLT, right-sided ligamentum teres. **b** Coronal contrast-enhanced CT showed the ligamentum teres observed on the right side of the gallbladder. GB, gallbladder; RSLT, right-sided ligamentum teres

**Fig. 3:**
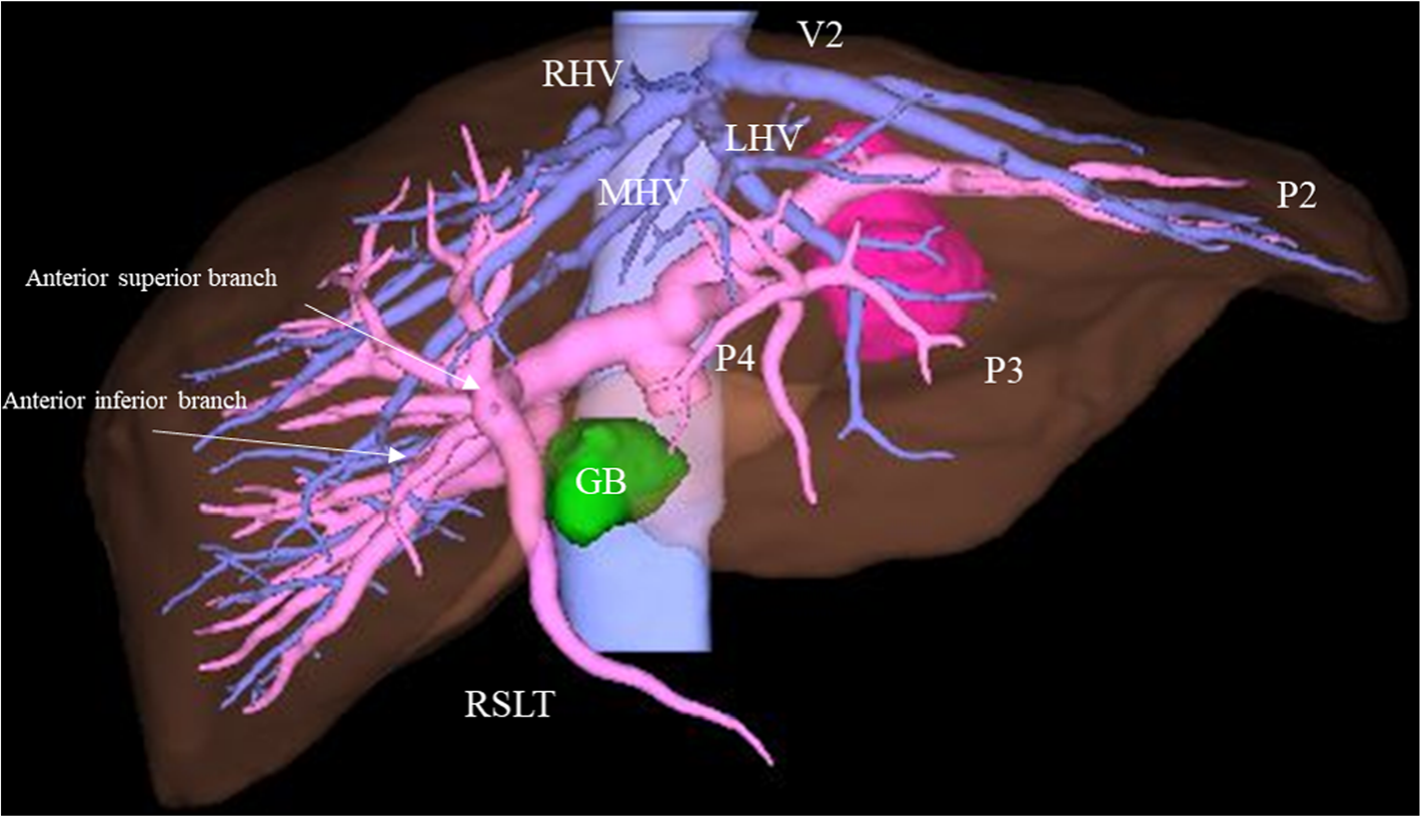
Three-dimensional CT clearly showed that the umbilical portion of the portal vein was located on the right anterior portal vein, where the RSLT connected. P2, portal vein branch of the left lateral superior section; P3, portal vein branch of the left lateral inferior section, P4, left paramedian branch of the portal vein, V2, drainage vein of the lateral superior section; RHV, right hepatic vein; MHV, middle hepatic vein; LHV, left hepatic vein; GB, gallbladder; RSLT, right-sided ligamentum teres

**Fig. 4:**
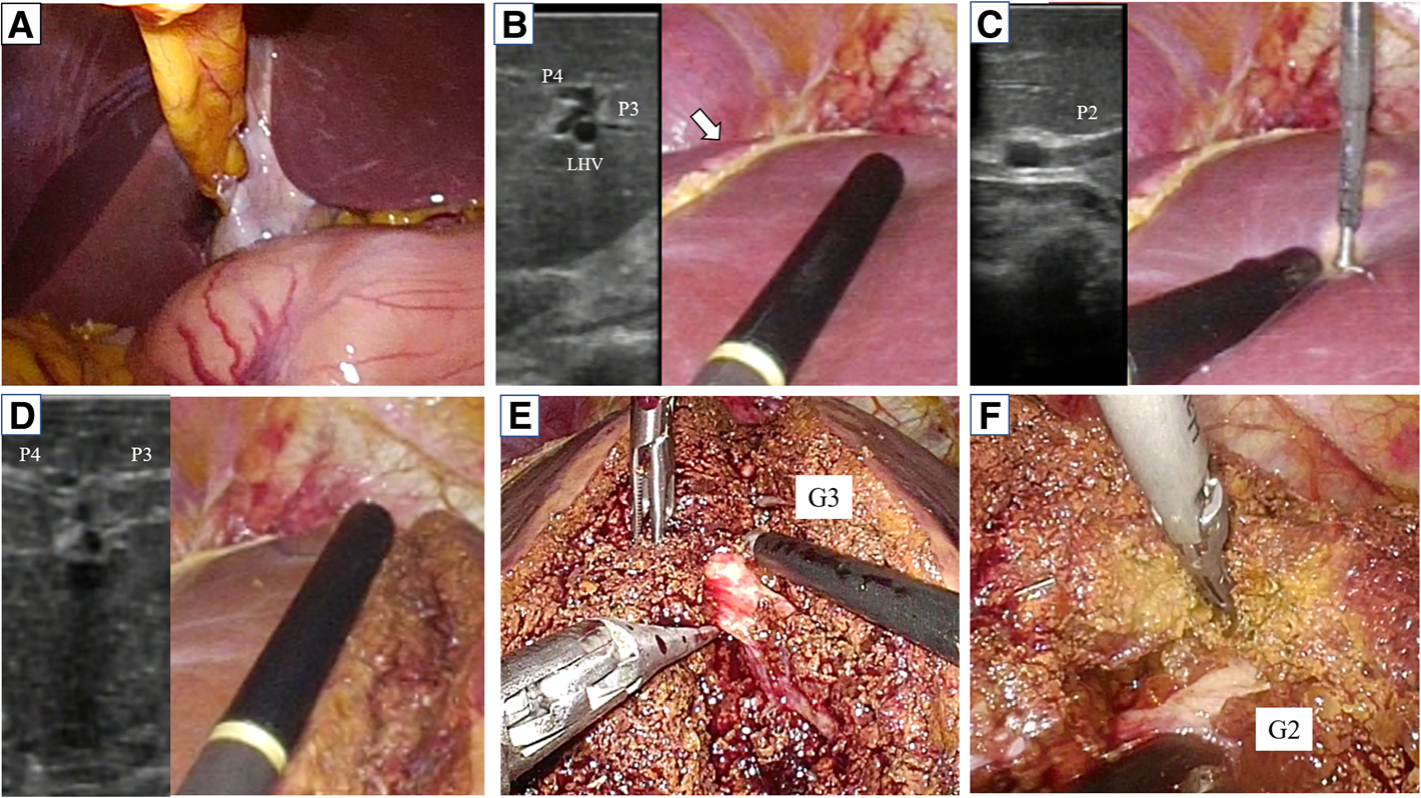
**a** The intraoperative findings included an RSLT with a left-sided gallbladder. **b** The resection line was carefully decided by identifying the portal vein branch of the lateral inferior section (P3) and left paramedian section (P4) using intraoperative ultrasonography. The falciform ligament is indicated by an arrow. LHV, left hepatic vein. **c** The resection line was carefully decided by identifying the portal vein branch of the lateral superior section (P2).**d** IOUS was repeatedly used to ensure the resection of the lateral inferior branch (P3) of the portal vein. P4, the left paramedian branch of portal vein. **e**, **f** The lateral inferior branch (G3) and lateral superior branch (G2) of Glisson’s sheath were separately clipped and resected

A 63-year-old man with a past history of rectal cancer was admitted to our hospital for the treatment of CRLM. The patient had undergone laparoscopic high anterior resection to treat rectal cancer 7 months previously. The pathological stage of the rectal cancer was T1N1aM0 stage IIIA, according to the Union for International Cancer Control classification (seventh edition). The patient did not experience any perioperative complications. He refused to receive adjuvant chemotherapy. Computed tomography (CT) performed 7 months after primary surgery revealed liver metastasis in the left lateral section. Indocyanine green retention at 15 min (ICGR15) was 4.1%. The patient’s Child-Pugh classification was class A. Tests for hepatitis B virus surface antigen and antibodies against hepatitis C virus were negative. Abdominal contrast-enhanced CT revealed a hypovascular tumor of 38 mm in size in the left lateral section (Fig. 1). The ligamentum teres was observed on the right side of the gallbladder (Fig. 2a, b). Three-dimensional CT clearly showed that the umbilical portion of the portal vein was located on the right anterior portal vein, where the RSLT connected (Fig. 3). Considering the segmentation of the liver according to the Brisbane 2000 terminology [4], the right anterior branch of the portal vein was ramified from the right portal vein. The lateral superior branch of the portal vein (P2) branched off behind the bifurcation of the portal vein, running separately from the common branch of the lateral inferior branch (P3) and left paramedian branch (P4). The patient was diagnosed with CRLM, and LLLS was planned. We fixed the patient in the supine and open-leg position and inserted four ports. The intraoperative findings showed an RSLT with a left-sided gallbladder (Fig. 4a). After mobilizing the left lateral section, the resection line was carefully decided by identifying the origin of the P3 and P4 of the portal vein using intraoperative ultrasonography (IOUS) (Fig. 4b). The P2 was independently ramified from the left portal vein and was identified by IOUS (Fig. 4c). The resection line was on the left side of the falciform ligament. Liver resection was performed using an ultrasonic scalpel (Harmonic Scalpel; Ethicon, CO) and repeatedly using IOUS to ensure the resection of the lateral superior branch of Glisson (G2) and lateral inferior branch of Glisson (G3) (Fig. 4d). The G3 and the G2 had to be separately clipped and resected, because of the wide distance between the two branches on the resection line (Fig. 4e, f). The vein draining the left lateral superior section (V2) and left hepatic vein (LHV) were resected at the cut surface, and the left paramedian branch of Glisson (G4) was correctly preserved using intraoperative ultrasonography. The operation time was 2 h and 58 min and the intraoperative blood loss was 50 g. The patient had a favorable clinical course without any complications and was discharged on postoperative day 5.

## Discussion

This is the first case report to mention the surgical strategy for laparoscopic left lateral sectionectomy (LLLS) for a patient with RSLT. In comparison to other liver resection procedures, LLLS has become a simple operation for patients with a normal anatomy due to the simple anatomy of the left lateral section and technical progression in laparoscopic devices. En bloc stapling of the G2 and G3 has helped to reduce the difficulty of LLLS in patients with a normal anatomy by reducing the operation time and blood loss [5]. In patients with a normal anatomy, LLLS can be simply performed but by stapling the G2 and G3 together on the left side of the umbilical portion (UP), because the P2 and P3 obviously ramify from the umbilical portion on the left side. Thus, it is not necessary to identify and clip the G2 and G3 separately. In contrast, en bloc stapling of the G2 and G3 is not a suitable choice in patients with an RSLT. The origins of the P2 and P3 cannot be recognized based on the appearance of the liver alone and are difficult to identify without using IOUS. The falciform ligament is not a landmark of the origin of the G2 and G3, which are buried in the liver. Frequent use of IOUS was important to recognize the P2, P3, and P4 and to determine the resection line. In our patient, there was a wide distance between the G2 and G3; two branches had to be separately resected. Operative ingenuity is necessary to avoid the misidentification of anatomical variation and safely perform LLLS for the patients with an RSLT. If LLLS is planned for patients with an RSLT and the variation of RSLT is not recognized before surgery, left hepatectomy may be carried out under the mistaken impression that the resection line should be along the falciform ligament. To avoid this mistake in patients with an RSLT, it is important to correctly recognize the RSLT on preoperative imaging.

When treating patients with RSLT, surgeons should understand that portal vein and hepatic vein deviation are not rare [6]. The posterior branch of the portal vein often ramifies independently and the anterior branch ramifies from the lateral branch in some cases [7–9]. Yamashita et al. [8] showed portal vein anomalies in 14 patients with RSLT. In nine of these cases, they did not have a transverse portion and umbilical portion but had a straight lateral branch of the portal vein that branched off P2, P3, and a thinner P4. The other five cases had a left portal vein that seemed to have a usual transverse portion, while the umbilical portion through the anterior branch was ramified from the tip of the left portal vein. The patients with a transverse portion and umbilical portion represented a minority group; in most cases, patients with RSLT only have a lateral branch of the portal vein.

The hepatic vein should be identified from the position of Rex-Cantlie line, which is shifted to the left side [10]. Each section should be defined based on the correlation between the portal vein and the drainage vein. In our case, the P2 was firstly ramified from the portal trunk proximal to the posterior branch of the portal vein, and secondly, the right portal vein and the P3 and P4 were ramified. We firstly regarded the leftmost vein as the V2 considering that this area was mainly fed by the P2. We next regarded the second left-sided vein as the LHV, which drained the section fed by the P2, P3, and P4.

Previous reports on liver resection for malignant disease in patients with an RSLT [11–15] are shown in Table 1. Modified hemihepatectomy was performed in all of the previous studies, which mainly reported the method for handling the bile duct of the right and left medial sections because the bile duct communication between the right and left medial section is often recognized in patients with an RSLT and it is necessary to prevent bile leakage after hemihepatectomy. At this time, we showed the operative ingenuity for LLLS to understand vasculobiliary anomaly in patients with RSLT and to prevent misidentification of the branches.

**Table 1:** Previous reports of liver resection for malignant disease in patients with RSLT

No.	Author	Year	Disease	Type of Hx	Operation time (min)	Blood loss (g)	Complication
1	Kaneoka et al. [11]	2000	ICC	Left hemiHx	Unknown	Unknown	–
2	Kaneoka et al. [11]	2000	ICC	HPD	Unknown	Unknown	–
3	Abe et al. [12]	2012	CRLM	Right hemiHx	526	750	–
4	Almodhaiberi et al. [13]	2015	Hilar cholangiocarcinoma	Left hemiHx	Unknown	Unknown	–
5	Hai et al. [14]	2017	Hilar cholangiocarcinoma	Extended left Hx	Unknown	Unknown	Bile leakage
6	Goto et al. [15]	2018	Gallbladder cancer	Right hemiHx	682	430	–
7	Our case	2018	CRLM	LLLS	178	50	–

*RSLT* right-sided ligamentum teres, *ICC* intrahepatic cholangiocarcinoma, *CRLM* colorectal liver metastasis, *HPD* hepato-pancreatoduodenectomy, *Hx* hepatectomy, *LLLS* laparoscopic left lateral sectionectomy

## Conclusions

In patients with an RSLT, the falciform ligament is not a landmark of the origins of the G2 and G3, which are buried in the liver and difficult to identify without IOUS. In the present case, these two branches had to be separately resected. The recognition of the RSLT on preoperative imaging is important for avoiding the mistaken impression that the resection line should be along the falciform ligament.
